# On the Connection between Puerperal Fever and Epidemic Erysipelas, in Its Origin and Mode of Propagation

**Published:** 1846-06-01

**Authors:** 


*On the Connection between Puerperal Fever and Epidemic Erysipelas, in its Origin and mode of Propagation.*

This is the title of an able article in the April number of the *American Journal of the Medical Sciences*, from the pen of Samuel Kneeland, Jr., M. D., of Boston. The writer sums up the conclusions at which he arrives in five propositions, which we quote, referring our readers to the article for the authorities and arguments by which they are substantiated. These propositions seem to us to be legitimate deductions from existing facts, and to express the views generally entertained on the subject by intelligent practitioners.

"1. That puerperal fever is not a common peritonitis, but a general diseased condition, in which the peritoneum, and every structure of the uterus, singly or jointly, may be affected; according as one or the other is attacked, giving rise to a series of symptoms, as complications, which may impose upon the observer as the real disease, and add to the gravity of the affection according to the organ diseased—thus explaining the contradictory statements of authors.

"2. Erysipelas is also dependent on a diseased condition of the whole system, and may manifest its action externally on the skin, or internally on any viscus, or membranous structure; the skin and cellular tissue are most frequently the seats of the affection—the serous membranes being a modification of the latter; and the peritoneum being predisposed by gestation and parturition, explains the frequent affection of these in the abdomen.

"3. The same seasons, and the same morbid states of the atmosphere give rise to puerperal fever and erysipelas; from their almost constant co-existence, their common origin is evident, though the intimate nature of the cause be unknown.

"4. The same morbid poison gives rise to these two diseases; in other words, puerperal fever is often only erysipelas attacking a puerperal woman; presenting the same constitutional symptoms, march, and morbid appearances, with a tendency to the uterine organs, as would be naturally expected. [We do not mean to say that puerperal fever is synonymous with erysipelas, as we would not quarrel with those who may persist in calling by this name intestinal irritation, inflammatory fever, and the many inflammations from common injuries during labour or the puerperal state ; or, in fine, any suspicious disease, occurring at such a time, and in want of a name."]

"5. The practitioner should not attend, or dress, cases of erysipelas when attending women in labour ; and should much less make autopsies of such cases."

"In conclusion we may remark, we conceive it to have been shown that puerperal fever is often contagious, and the erysipelatous form remarkably so ; and that these two diseases have the same origin, one and the same contagion operating in the production of both."

